# In vivo Efficacy and Safety Evaluation of Lactosyl-β-cyclodextrin as a Therapeutic Agent for Hepatomegaly in Niemann-Pick Type C Disease

**DOI:** 10.3390/nano9050802

**Published:** 2019-05-25

**Authors:** Yuki Maeda, Keiichi Motoyama, Rena Nishiyama, Taishi Higashi, Risako Onodera, Hideaki Nakamura, Toru Takeo, Naomi Nakagata, Yusei Yamada, Yoichi Ishitsuka, Yuki Kondo, Tetsumi Irie, Takumi Era, Hidetoshi Arima

**Affiliations:** 1 Graduate School of Pharmaceutical Sciences, Kumamoto University, Kumamoto 862-0973, Japan; maedayuki.jpn@gmail.com (Y.M.); 2525r.n.c@gmail.com (R.N.); higashit@kumamoto-u.ac.jp (T.H.); 162y3101@st.kumamoto-u.ac.jp (Y.Y.); y-zuka@gpo.kumamoto-u.ac.jp (Y.I.); ykondo@kumamoto-u.ac.jp (Y.K.); tirie@gpo.kumamoto-u.ac.jp (T.I.); 2 Program for Leading Graduate Schools “HIGO (Health life science: Interdisciplinary and Glocal Oriented) Program”, Kumamoto University, Kumamoto 862-0973, Japan; 3 Priority Organization for Innovation and Excellence, Kumamoto University, Kumamoto 862-0973, Japan; 4 School of Pharmacy, Kumamoto University, Japan; deragon@kumamoto-u.ac.jp; 5 Faculty of Pharmaceutical Sciences, Sojo University, Kumamoto 860-0082, Japan; nhideaki@ph.sojo-u.ac.jp; 6 Center for Animal Resources and Development, Kumamoto University, Kumamoto 860-0811, Japan; takeo@kumamoto-u.ac.jp (T.T.); nakagata@kumamoto-u.ac.jp (N.N.); 7 Department of Cell Modulation, Institute of Molecular Embryology and Genetics, Kumamoto University, Kumamoto 860-0811, Japan; tera@kumamoto-u.ac.jp

**Keywords:** Cyclodextrins, cholesterol, Niemann-Pick Type C disease, hepatomegaly, liver targeting

## Abstract

Niemann-Pick type C disease (NPC) is a fatal, autosomal recessive disorder, which causes excessive accumulation of free cholesterol in endolysosomes, resulting in progressive hepatomegaly and neurodegeneration. Currently, 2-hydroxypropyl-β-cyclodextrin (HP-β-CyD) is used at a high dose for the treatment of NPC, risking lung toxicity and hearing loss during treatment. One method to reduce the required dose of HP-β-CyD for the treatment of hepatomegaly is to actively deliver β-cyclodextrin (β-CyD) to hepatocytes. Previously, we synthesized lactosyl-β-CyD (Lac-β-CyD) and demonstrated that it lowers cholesterol in NPC model liver cells. In the present study, we studied the efficacy and safety of Lac-β-CyD treatment of hepatomegaly in *Npc1*^−/−^ mice. After subcutaneous administration, Lac-β-CyD accumulated in the liver and reduced hepatomegaly with greater efficacy than HP-β-CyD. In addition, subcutaneous administration of a very high dose of Lac-β-CyD was less toxic to the lungs than HP-β-CyD. Notably, the accumulation of intracellular free cholesterol in endolysosomes of NPC-like liver cells was significantly lower after administration of Lac-β-CyD than after treatment with HP-β-CyD. In conclusion, these results suggest that Lac-β-CyD is a candidate for the effective treatment of hepatomegaly in NPC.

## 1. Introduction

Lysosomal storage diseases are congenital metabolic disorders caused by an absence or abnormality of lysosomal enzymes or proteins [1]. These diseases are inherited in an autosomal-recessive fashion and are characterized by the abnormal accumulation of lipids, glycoproteins, and mucopolysaccharides in lysosomes [2]. The disorders are classified into seven types according to the accumulated substrates: (1) glycolipid metabolism disorder (sphingolipidosis); (2) mucopolysaccharide metabolism disorder (mucopolysaccharidosis); (3) glycoprotein metabolism disorder; (4) mucolipidosis; (5) glycogen storage disease type II; (6) acid lipase deficiency; and (7) lysosomal membrane protein disorder [3,4].

Niemann-Pick disease type C (NPC) is a lysosomal storage disease that is characterized by the accumulation of free cholesterol and glycosphingolipids in endolysosomes due to the loss of membrane protein, NPC1, or soluble protein, NPC2. Mutations in the *NPC1* gene are observed in approximately 95% of NPC patients, with the remainder having mutations in the NPC2 gene [5]. The incidence of NPC is approximately 1 in 150,000 live births. Enlargement of the liver (hepatomegaly) or spleen (splenomegaly) is observed in around 85% of NPC patients in childhood to early adolescence, accompanied by abdominal and lower back pressure and pain. In addition, the abnormal accumulation of free cholesterol and glycosphingolipids in brain tissue of NPC patients leads to damage of brain tissue and causes various neurological symptoms. The majority of cases (60–70%) are classic NPC (late infantile and juvenile), and death generally occurs between 7 and 12 years of age [6]. Several approaches for the treatment of NPC have been proposed, such as glucosylceramide synthase inhibitors (i.e. miglustat) [7], histone deacetylase (HDAC) inhibitors (i.e. vorinostat) [8], curcumin [9], and 2-hydroxypropyl-β-cyclodextrin (HP-β-CyD) [10,11]. Unfortunately, the efficacies of miglustat, vorinostat, and curcumin are so limited that patients eagerly await new therapies for NPC. Hence, HP-β-CyD has received tremendous attention as a potential NPC therapeutic agent due to its high efficacy, although a high dose is required.

Cyclodextrins (CyDs) are cyclic oligosaccharides with a hydrophobic cavity that can form inclusion complexes with various guest molecules. HP-β-CyD has been most widely used in the pharmaceutical field to improve drug solubility and bioavailability. It has been reported that HP-β-CyD removes cholesterol from lipid rafts on the plasma membrane surface and destroys the structures [12,13]. In addition, subcutaneous administration of HP-β-CyD to NPC model mice decreased free cholesterol levels, ameliorated hepatomegaly, and delayed the progression of neurological symptoms (10). Phase I/II and phase IIb/III clinical trials of HP-β-CyD administered intravenously (CTD holdings, Inc.) and intrathecally (Mallinkrodt plc.), respectively, are ongoing. In particular, the administration into the space under the arachnoid membrane of the brain (intrathecal administration) of HP-β-CyD has shown excellent therapeutic effects. However, to obtain efficacy in vivo, high-dose administration of HP-β-CyD is necessary, as HP-β-CyD enter cells only very slightly, owing to its hydrophilicity and relatively high molecular weight. Indeed, Matsuo et al. reported that an NPC patient exhibited fever and transient cloudiness of the lungs after two years of treatment with HP-β-CyD by intravenous infusion. Therefore, the administration route was altered to intraventricular administration [14].

Although HP-β-CyD is clinically applied to NPC treatment, HP-β-CyD has no cell or tissue selectivity. Therefore, the application of CyD having liver selectivity is expected for the treatment of hepatosplenomegaly in NPC. Meanwhile, asialoglycoprotein receptor (ASGPR), a hepatic galactose and N-acetylglucosamine (GlcNAc) receptor, is responsible for the binding, internalization, and subsequent clearance of glycoproteins containing terminal galactose or GlcNAc residues from the circulation [15]. Hence, galactosylated nanocarriers have been used for selective delivery of drugs to the liver via ASGPR-mediated endocytosis [16]. In fact, ASGPR-mediated endocytosis is one of the most promising approaches for delivery of CyDs into hepatocytes for the treatment of hepatosplenomegaly in NPC disease. In our previous report, mono-lactosyl β-CyD (mono-Lac-β-CyD) diminished cholesterol accumulation as efficiently as HP-β-CyD in our model of NPC hepatocytes, U18666A-treated HepG2 liver cells (NPC-like HepG2 cells: U18666A ((3β)-3-[2-(Diethylamino)ethoxy]androst-5-en-17-one hydrochloride) is an inhibitor of lysosomal cholesterol export [17]. ASGPR recognizes the galactose moiety at three points, collectively known as the “golden triangle” [18,19]. However, as mono-Lac-β-CyD can bind to only one point of the golden triangle of ASGPR, it has insufficient binding affinity for ASGPR-mediated internalization. Recently, we created a novel multi-lactosyl β-CyD (Lac-β-CyD) to improve targeting to ASGPR, and evaluated its cholesterol-lowering effect in NPC-like HepG2 cells [20]. Lac-β-CyD was internalized into hepatocytes via ASGPR-mediated endocytosis and ameliorated excessive accumulation of free cholesterol in NPC-like HepG2 cells. However, its therapeutic effect on hepatomegaly in vivo has not yet been reported.

Based on the above considerations, we aimed to examine the therapeutic effect of Lac-β-CyD on hepatomegaly in the NPC model mice (*Npc1*^−/−^ mice), and to evaluate its safety in wild type (WT) mice. Initially, we used fluorescently-labeled Lac-β-CyD and HP-β-CyD to investigate their accumulation in the liver after subcutaneous administration to WT mice. Secondly, we examined the effects of Lac-β-CyD on vacuolar degeneration of the liver, liver inflammation, and tissue cholesterol accumulation in *Npc1*^−/−^ mice after single or weekly administration of Lac-β-CyD. Finally, we analyzed plasma biochemistry and studied the lung toxicity of high acute doses of Lac-β-CyD and HP-β-CyD in WT mice. The results suggested that Lac-β-CyD has great potential for safer and effective treatment of hepatomegaly in NPC.

## 2. Materials and Methods 

### 2.1. Materials

Lac-β-CyD with average degree of substitution of lactose of 5.6 was fabricated as reported previously [20]. HP-β-CyD was kindly donated by Nihon Shokuhin Kako (Tokyo, Japan). HyLite^TM^ Fluor 647 (HF647) acid, SE, was obtained from Anaspec, Inc. (Fremont, CA, USA). Asialofetuin (AF) from fetal calf serum Type I and bovine serum albumin (BSA) Fraction V were purchased from Sigma-Aldrich (St. Louis, MO, USA). The BioRad protein assay kit, CellLight^®^ ER-GFP BacMam 2.0, LysoTracker^®^ Green DND-26 (LysoTracker^®^), and LysoTracker^®^ Red DND-99 (LysoTracker^®^) were purchased from Thermo Fisher Scientific, Inc. (Waltham, MA, USA). U18666A, maltoheptaose, miglustat hydrochloride, and Cholesterol E-test Wako^®^ were purchased from Wako Pure Chemical Industries (Osaka, Japan). Filipin III was purchased from Cayman Chemical (Ann Arbor, MI, USA). Hoechst 33342, and secondary antibodies, goat anti-mouse IgG Alexa Fluor^®^ 488 (A11029) and goat anti-rabbit IgG Alexa Fluor^®^ 594 (A11037), were obtained from Invitrogen (Carlsbad, CA, USA). Cyto-ID^TM^ Autophagy Detection Kit was obtained from Enzo Life Sciences (Farmingdale, NY, USA). Anti-TFE3 antibody (#4240) was obtained from Cell Signaling Technology (Danvers, MA, Japan). SQSTM1 (sc-28359) was obtained from Santa Cruz Biotechnology (Dallas, TX, USA). The cell-counting kit-8 was obtained from Dojindo Laboratories (Kumamoto, Japan). Other chemicals and solvents were of analytical reagent grade.

### 2.2. Synthesis of HF647-Labeled β-CyDs

As we previously reported, we synthesized and characterized fluorescently-labeled β-CyDs [17,20]. Briefly, to introduce amino groups into HP-β-CyD for the synthesis of fluorescently-labeled HP-β-CyD, HP-β-CyD (100 mg, 71.9 μmol), 1,1-carbonyldiimidazol (98 mg, 604 μmol), and triethylamine (20 μL, 0.14 μmol) were dissolved in 1.2 mL of dehydrated DMSO and stirred for 1.5 h at room temperature. Ethylenediamine (6.8 μL, 1.0 μmol) was added to the reaction mixture and the solution was stirred for a further 5 h at room temperature under a nitrogen atmosphere. After the reaction, NH_2_-HP-β-CyD was purified by the acetone precipitation method: the sample was gradually dropped into 50 mL of acetone. After centrifugation (10,000 rpm, 10 min), the precipitant was collected and dissolved in water. The recovered solution was freeze-dried to obtain NH_2_-HP-β-CyD as powder. To synthesize HF647-labeled β-CyDs, Lac-β-CyD (100 mg, 34 μmol) and NH_2_-HP-β-CyD (100 mg, 72 μmol) were dissolved in 10 mL and 1.5 mL of dehydrated DMSO, respectively. HF647 acid (500 μg, 384 nmol) was added to each reaction mixture and the solutions were stirred in the dark for 24 h at room temperature. HF647-labeled Lac-β-CyD and HF647-labeled HP-β-CyD were purified by the acetone precipitation method.

### 2.3. Cell Culture

The human hepatocellular carcinoma cell line, HepG2, was cultured in Dulbecco’s Modified Eagle’s Medium (DMEM, Nissui Pharmaceuticals, Tokyo, Japan), containing penicillin (1 × 10^5^ mU/mL) and streptomycin (0.1 mg/mL), and supplemented with 10% fetal bovine serum (FBS), in a humidified incubator at 5% CO_2_. NPC-like HepG2 cells containing high amounts of accumulated cholesterol were obtained after 48 h incubation with DMEM containing 1.25 μM U18666A.

### 2.4. Mice

Male and female homozygous (*Npc1*^−/−^) mutant (BALB/cNctr-*Npc1*^m1N^) mice were kindly donated by Dr. Kosaku Ohno and Dr. Katsumi Higaki. Wild-type (WT) BALB/c mice were obtained from Japan SLC, Inc. (Shizuoka, Japan). The mice were bred and kept in specific pathogen-free conditions in the Center for Animal Resources and Development, Kumamoto University. All animal experiments in this manuscript were carried out in accordance with the guidelines approved by the Ethics Committee for Animal Care and Use of Kumamoto University composed of a third party (Approval ID: A29-125).

### 2.5. In Vivo Fluorescence Imaging

The concentration of each of the HF647-β-CyDs in the serum and various organs, including the heart, lung, liver, kidney and spleen, was measured to evaluate the pharmacokinetics of Lac-β-CyD and HP-β-CyD after subcutaneous administration to *Npc1*^−/−^ mice. Briefly, HF647-Lac-β-CyD or HF647-HP-β-CyD in PBS was administered to 8-week-old WT mice by subcutaneous injection, at a dose of 20 mg/kg or 10 mg/kg, respectively. To study the effect of ASGPR on the distribution of HF647-Lac-β-CyD, 500 mg/kg AF, a competitive inhibitor of ASGPR, was intraperitoneally administered to the mice, 1 min before and 5 min after administration of the labeled β-CyDs. An hour after administration of the test compounds, the mice were sacrificed and perfused with PBS. The organs were harvested, and fluorescent images of the organs were acquired using an IVIS Lumina XRMS in vivo Imaging System (Perkin Elmer Inc., Waltham, MA, USA). 

### 2.6. Treatment of NPC Model Mice with CyDs

Lac-β-CyD or HP-β-CyD was dissolved in PBS to 38 mM, and the solution was filtered using DISMIC^®^-13HP 0.20 μm (Advantec, Toyo Roshi, Tokyo, Japan). For the acute study, either Lac-β-CyD (0.7 mmol/kg, 2098 mg/kg) or HP-β-CyD (0.7 mmol/kg, 1000 mg/kg) was administered to WT and *Npc1*^−/−^ mice (8 week old) by subcutaneous injection (injection volume: 20 μL/g of body weight). The mice were anesthetized 24 h after dosing, and the blood and organs were collected. To study the effect of repeated dosing of the animals, Lac-β-CyD (0.7 mmol/kg, 2098 mg/kg) or HP-β-CyD (0.7 mmol/kg, 1000 mg/kg) was administered by subcutaneous injection at the same site to WT and *Npc1*^−/−^ mice once a week from 4 to 8 weeks of age (injection volume: 20 μL/g of body weight). Blood and various organs were collected as described above 72 h after the final dose.

### 2.7. Biochemical and Histological Analysis*β*

Levels of aspartate aminotransferase (AST) and alanine aminotransferase (ALT) in blood were determined using a FUJI DRI-CHEM 7000 automated clinical chemistry analyzer (FUJIFILM, Tokyo, Japan). After collection of the blood, the mice were infused with saline, and the brain, lungs, liver, spleen, and kidneys were harvested. The tissues were fixed with 10% neutral buffered formalin before being embedded in paraffin. The paraffin blocks were sectioned and stained with hematoxylin and eosin (H&E). For histological analysis, the sections were analyzed using a Biorevo BZ-9000 (Keyence, Osaka, Japan). To evaluate cholesterol levels in liver tissue, the liver samples were fixed in 4% paraformaldehyde (PFA) solution for 48 h at 4 °C. After 3 washes in PBS, the liver samples were incubated overnight in 30% sucrose in PBS, sectioned, and embedded in Tissue-Tek^®^ O.T.C. Compound (Sakura Finetek, Tokyo, Japan) before freezing. The frozen sections were incubated in 1% BSA for 1 h at room temperature, and stained with 50 μg/mL of Filipin III solution (freshly dissolved in ethanol to 5 mg/mL and diluted in PBS) for 1 h at room temperature. After washing in PBS, the fluorescence derived from Filipin III in the liver sections was detected using confocal laser scanning microscopy (Leica TCS SP8, Leica, Wetzlar, Germany).

### 2.8. In Vivo Safety Evaluation of CyDs in WT Mice

Lac-β-CyD (8 mmol/kg, 23,632 mg/kg) or HP-β-CyD (8 mmol/kg, 11,120 mg/kg) was administered subcutaneously to WT mice (8 weeks old). The lungs, liver, and kidneys were collected 8 h later. To study the toxicity of the compounds to the lungs, the number of inflammatory cells and total protein concentration in the bronchoalveolar lavage fluid (BALF) were determined. Histological analysis of H&E-stained lung, liver, and renal tissues was performed to study the effects of the compounds on these tissues. In addition, the amounts of AST, ALT, blood urea nitrogen (BUN), and lactate dehydrogenase (LDH) in the blood were determined using a FUJI DRI-CHEM 7000 automated clinical chemistry analyzer (FUJIFILM, Tokyo, Japan).

### 2.9. Cellular Uptake Analysis 

U18666A-treated HepG2 cells (5 × 10^4^ cells/35 mm glass bottom dish) were transfected with ER-GFP BacMam 2.0 reagent, to label endoplasmic reticulum (ER) with green fluorescent protein (GFP), for 16 h. After 3 washes in PBS, the cells were exposed to culture medium containing HF647-β-CyDs (100 μM), in the presence or absence of AF (1.0 mg/mL), for 24 h. After washing in PBS, the cells were stained with 50 nM LysoTracker^®^ Red DND-99 (to label acidic organelles) for 30 min at 37 °C. After washing in PBS, the fluorescence derived from HF647, ER-GFP, and LysoTracker^®^ in U18666A-treated HepG2 cells was detected using confocal laser scanning microscopy (Leica TCS SP8, Leica, Wetzlar, Germany). The fluorescence intensity was quantified using ImageJ software.

### 2.10. Free Cholesterol Levels in Endolysosomes

U18666A-treated HepG2 cells (5 × 10^4^ cells/35 mm glass bottom dish) were incubated in culture medium containing 1 mM β-CyDs, miglustat, or maltoheptaose in the presence or absence of AF (1.0 mg/mL), for 24 h. After washing in PBS, the cells were stained using 50 nM LysoTracker^®^ Green DND-26 (to stain acidic cellular compartments) for 30 min at 37 °C. After washing in PBS, the cells were fixed in 4% PFA for 10 min at room temperature and stained with Filipin III (50 μg/mL), a fluorescent cholesterol-binding molecule, for 1 h at room temperature. After washing in PBS, the fluorescence derived from Filipin III and LysoTracker^®^ in the cells was detected using confocal laser scanning microscopy (Leica TCS SP8, Leica, Wetzlar, Germany). The fluorescence intensity was quantified using ImageJ software.

### 2.11. Analysis of Cholesterol Solubilization by CyDs 

In a phase solubility study, volumes of 0.2 mL of Lac-β-CyD or HP-β-CyD solution (0, 0.5, 1.0, 2.0, 2.5, 4.0, 5.0, 7.5, and 10 mM) were added to glass vials containing approximately 2 mg of cholesterol. The suspensions were vigorously shaken for 72 h at room temperature and filtered through a 0.20 μm DISMIC^®^-13HP to remove undissolved cholesterol. The cholesterol concentration was determined using a Cholesterol E-test Wako^®^ (Osaka, Japan).

### 2.12. Hemolytic Activity of CyDs in Rabbit Red Blood Cells

Rabbit red blood cells (RBCs) were isolated from Japanese white male rabbits (Kyudo, Tosu, Japan) as described previously [21,22]. Isolated RBCs were centrifuged at 1000 g for 5 min and washed 3 times in PBS. Aliquots of RBC suspension in PBS were incubated in 1 mL of Lac-β-CyD or HP-β-CyD (0.1 and 1.0 mM in PBS) for 30 min at 37 °C. After centrifugation at 1000 g for 10 min, the optical density of the supernatant was measured at 543 nm. The results were expressed as a percentage of the total hemolysis obtained when the RBCs were incubated in water only.

### 2.13. Cytotoxicity of CyDs 

Mitochondrial dehydrogenase activity was used to assess cell viability, and was measured using a water-soluble tetrazolium salt (WST-8) Cell Counting Kit-8 (Dojindo Laboratories, Kumamoto, Japan) according to the manufacturer’s protocol. U18666A-treated HepG2 cells (5 × 10^4^ cells/96 well plate) were treated in culture medium containing Lac-β-CyD or HP-β-CyD (at 10, 20, 30, and 40 mM) for 24 h, and then incubated in the WST-8 solution for 1 h at 37 °C. The absorbance at 450 nm was measured against a reference wavelength of 630 nm using a microplate reader (Bio-Rad iMark, Tokyo, Japan).

### 2.14. Data Analysis

Quantitative data are expressed as the mean ± standard error of the mean (S.E.M.), while the statistical comparisons were made using the Scheffe’s test. A *p*-value < 0.05 was considered statistically significant.

## 3. Results

### 3.1. In Vivo Fluorescence Imaging of HF647-Lac-β-CyD

**Figure 1:**
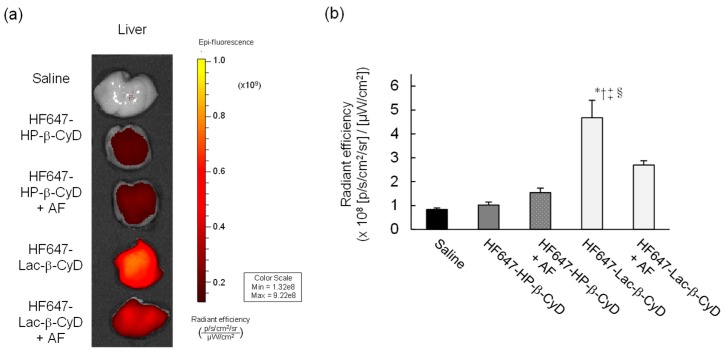
In vivo fluorescence imaging of HF647-Lac-β-CyD. (**a**) Liver accumulation of HF647-Lac-β-CyD and HF647-HP-β-CyD. The liver was harvested 60 min after subcutaneous administration of HF647-Lac-β-CyD (20 mg/kg) or HF647-HP-β-CyD (10 mg/kg) to WT mice, and fluorescence intensity of the liver was detected by IVIS. These figures show the representative images of 3 mice. (**b**) Effect of ASGPR on liver accumulation of HF647-Lac-β-CyD. AF (500 mg/kg) was intraperitoneally administered to WT mice 1 min before and 5 min after subcutaneous administration of HF647-Lac-β-CyD. After 60 min, the liver was harvested, and the fluorescence intensity was detected by IVIS.

To study the biodistribution of Lac-β-CyD after its subcutaneous administration to mice, we used an in vivo imaging system (IVIS) to monitor the fluorescence of HyLite™ Fluor 647-labeled Lac-β-CyD (HF647-Lac-β-CyD) and HF647-HP-β-CyD in several organs of the mice. One hour after administration, the accumulated level of HF647-Lac-β-CyD in the liver was significantly higher than that of HF647-HP-β-CyD (Figure 1a). HF647-Lac-β-CyD accumulated in the kidney, probably due to renal clearance (data not shown). Meanwhile, HF647-Lac-β-CyD showed negligible accumulation in heart, lung and spleen (data not shown). Next, we studied the effect of ASGPR on liver accumulation of subcutaneously administered HF647-Lac-β-CyD, in the presence or absence of AF, a competitor for ASGPR (Figure 1b). Importantly, liver accumulation of HF647-Lac-β-CyD was significantly lower in the presence of AF than in its absence. This suggests that Lac-β-CyD may accumulate in the liver through ASGPR-mediated endocytosis. On the other hand, the modification of HF 647 to Lac-β-CyD may have an influence on physical property and biodistribution. Therefore, it will be necessary to study biodistribution of Lac-β-CyD by another method without modification with fluorescence in the future.

### 3.2. Therapeutic Effect of Lac-β-CyD in NPC Mice

**Figure 2:**
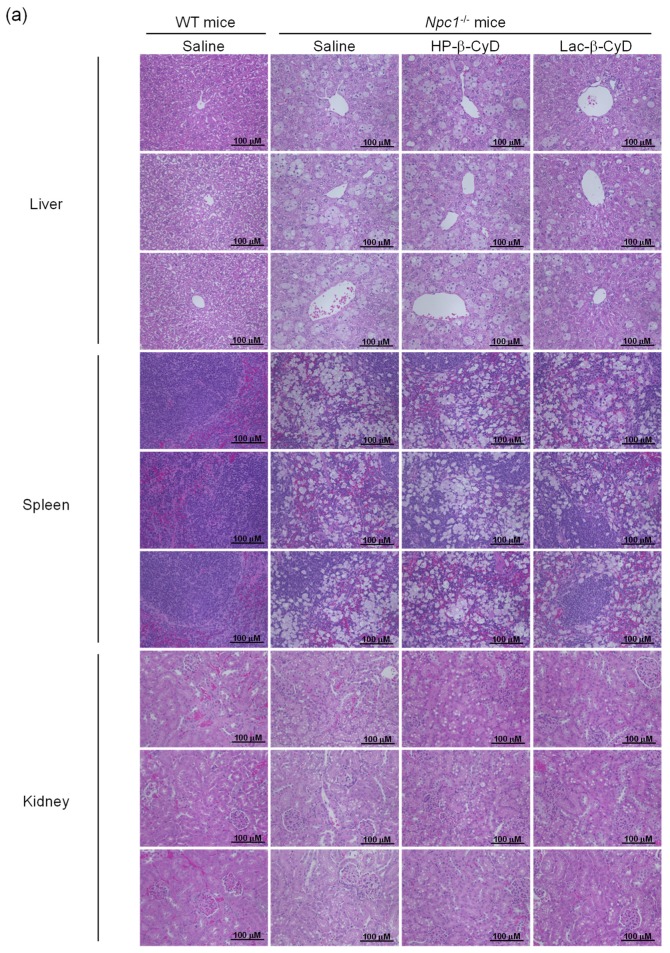

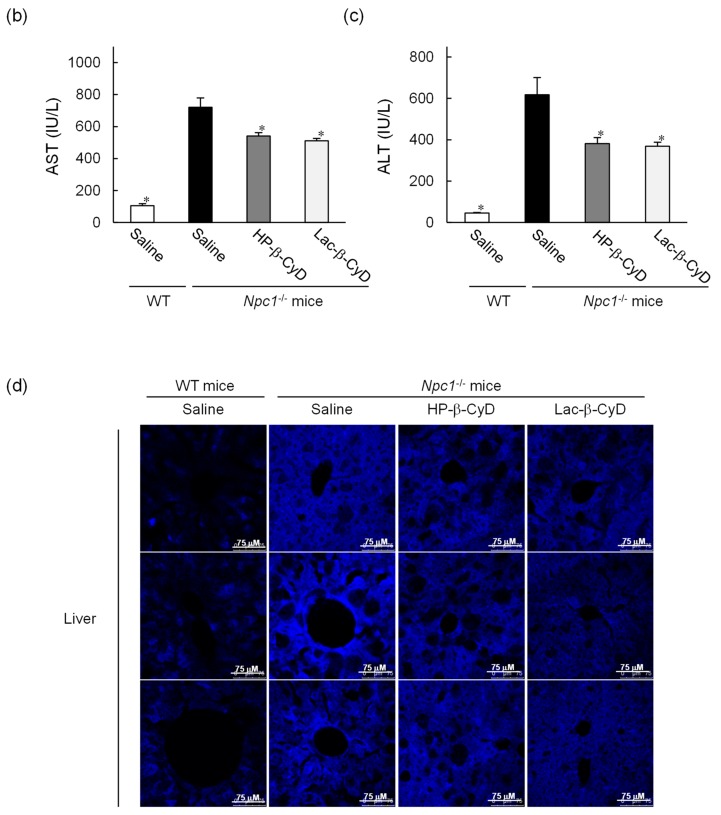
Therapeutic effects of Lac-β-CyD in NPC model mice after a single subcutaneous administration. Lac-β-CyD (0.7 mmol/kg, 2098 mg/kg), HP-β-CyD (0.7 mmol/kg, 1000 mg/kg), or saline was administered to 8 week old WT and Npc1^−/−^ mice via a single subcutaneous injection. The blood and organs were collected 24 h later. (**a**) Histological analysis of liver, spleen, and kidneys of the *Npc1*^−/−^ mice. These figures show the representative images of 3 different mice. (**b**,**c**) AST (b) and ALT (**c**) levels in plasma. Each value represents the mean ± S.E.M. of 4-7 experiments. * *p* < 0.05, compared to control *Npc1*^−/−^ mice. (**d**) Free cholesterol accumulation in the liver of the *Npc1*^−/−^ mice. These figures show the representative images of 3 different mice.

The NPC model mice (*Npc1*^−/−^) exhibit various NPC phenotypes, such as hepatomegaly, elevation of hepatic injury markers such as AST and ALT, and vacuolization of liver, spleen, and kidney tissues [10,11,23,24,25,26,27]. Therefore, we investigated the therapeutic effect of Lac-β-CyD on hepatomegaly after its subcutaneous administration to *Npc1*^−/−^ mice. Lac-β-CyD at a dose of 0.7 mmol/kg was subcutaneously administered to eight-week old *Npc1*^−/−^ mice (Figure 2). This 0.7 mmol/kg dose of Lac-β-CyD was a quarter of the effective dose of HP-β-CyD reported previously (2.8 mmol/kg, 4000 mg/kg).(23) As shown in Figure 2a, vacuolization and lipid-laden macrophages were observed in the livers of control *Npc1*^−/−^ mice, typical symptoms of hepatomegaly in NPC due to the accumulation of free cholesterol and glycolipids in liver. Dosing with Lac-β-CyD in liver, but not in spleen and kidney, markedly attenuated this morphology, to a far greater extent than after HP-β-CyD dosing (Figure 2a). In addition, a single subcutaneous administration of Lac-β-CyD markedly reduced AST and ALT levels in *Npc1*^−/−^mice (Figure 2b,c). Unfortunately, we were unable to find significant change in liver size or liver weight between Lac-β-CyD and HP-β-CyD under these experimental conditions. It might be necessary to optimize the administration protocol to see the difference in liver size or weight. However, most importantly, the fluorescence intensity of Filipin III, derived from free cholesterol, was significantly decreased in the livers of *Npc1*^−/−^ mice after a single subcutaneous dose of Lac-β-CyD, compared to that of control *Npc1*^−/−^ mice (Figure 2d). These results suggest that hepatomegaly is ameliorated in *Npc1*^−/−^ mice after a single subcutaneous dose of 0.7 mmol/kg of Lac-β-CyD. 

**Figure 3:**
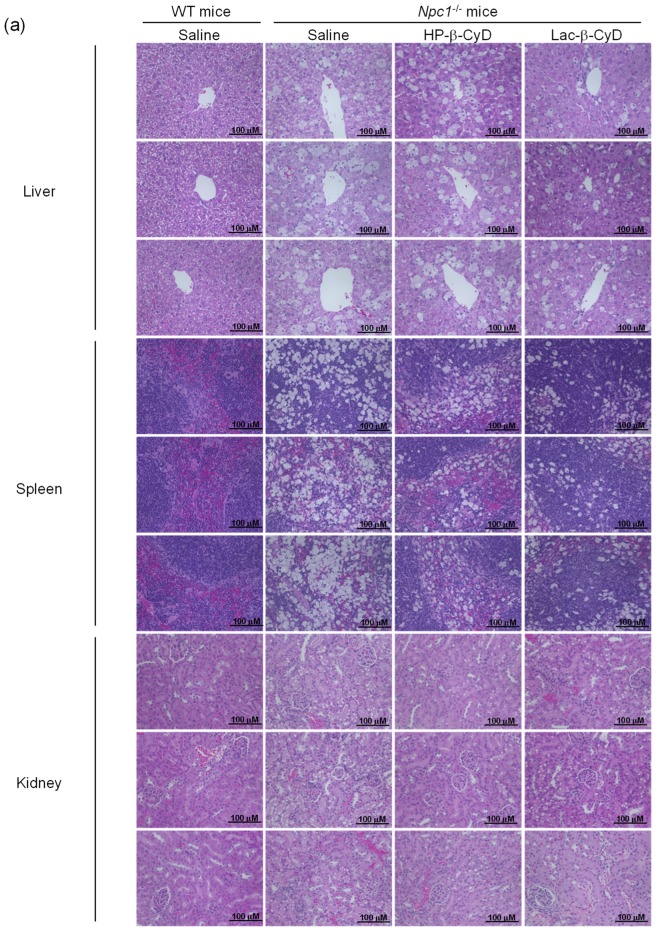

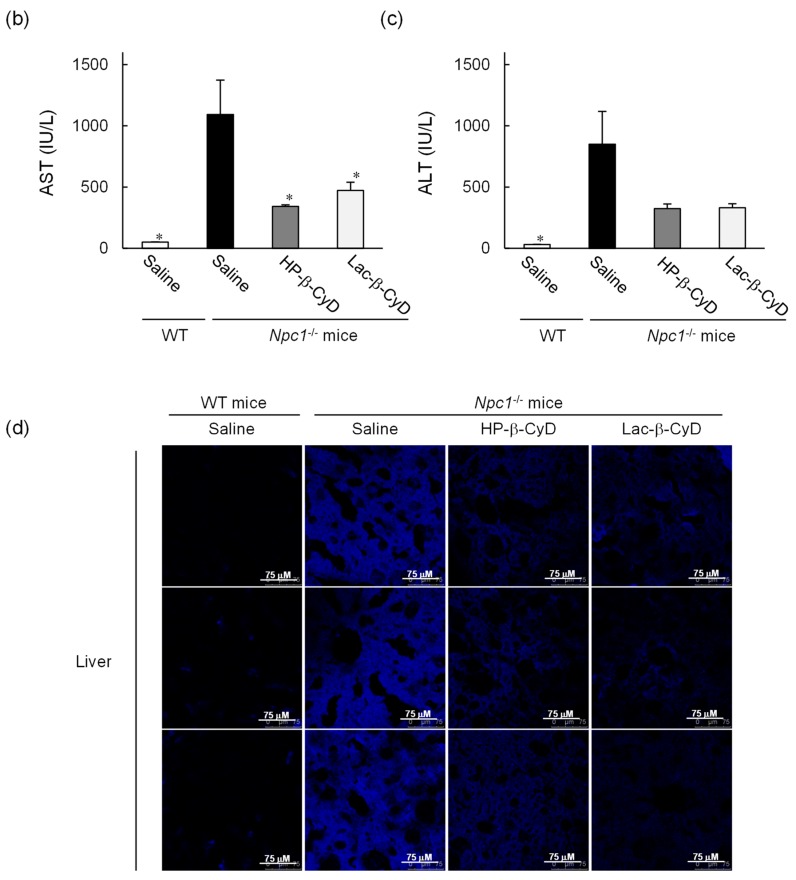
Therapeutic effects of once-weekly subcutaneous administration of Lac-β-CyD to NPC model mice. Lac-β-CyD (0.7 mmol/kg, 2098 mg/kg) or HP-β-CyD (0.7 mmol/kg, 1000 mg/kg) was subcutaneously administered to WT and *Npc1*^−/−^ mice once a week from 4 to 8 weeks, and the blood and organs were collected 72 h after the final injection. (**a**) Histological analysis of liver, spleen, and kidneys of *Npc1*^−/−^ mice. These figures show the representative images of three different mice. (B & C) AST (**b**) and ALT (**c**) levels in plasma. Each value represents the mean ± S.E.M. of 4-7 experiments. * *p* < 0.05, compared to control *Npc1*^−/−^ mice. (**d**) Free cholesterol accumulation in the liver of the *Npc1*^−/−^ mice. These figures show the representative images of three different mice.

To examine the effect of repeated dosing on the therapeutic effects, we administered Lac-β-CyD to 4 week old *Npc1*^−/−^ mice once a week from 4 to 8 weeks of age at a dose of 0.7 mmol/kg (Figure 3). Once weekly injection of Lac-β-CyD drastically reduced the vacuoles in the liver, spleen, and kidneys of *Npc1*^−/−^ mice, compared to that of HP-β-CyD (Figure 3a). In addition, weekly injections of Lac-β-CyD tended to reduce the AST and ALT levels (Figure 3b,c), and free cholesterol accumulation in the liver (Figure 3d). 

### 3.3. In Vivo Safety Evaluation of Lac-β-CyD in WT Mice 

Matsuo et al. reported that an intravenous infusion of HP-β-CyD was effective against hepatosplenomegaly in NPC patients, although one patient exhibited transient cloudiness of the lungs after two years of treatment [14]. Moreover, it has been reported that chronic HP-β-CyD infusion induced pneumonia in healthy pigs [28] and caused foamy macrophage infiltration in the lungs of rats [29], suggesting that there is a risk of lung toxicity when HP-β-CyD is used for NPC treatment.

**Figure 4:**
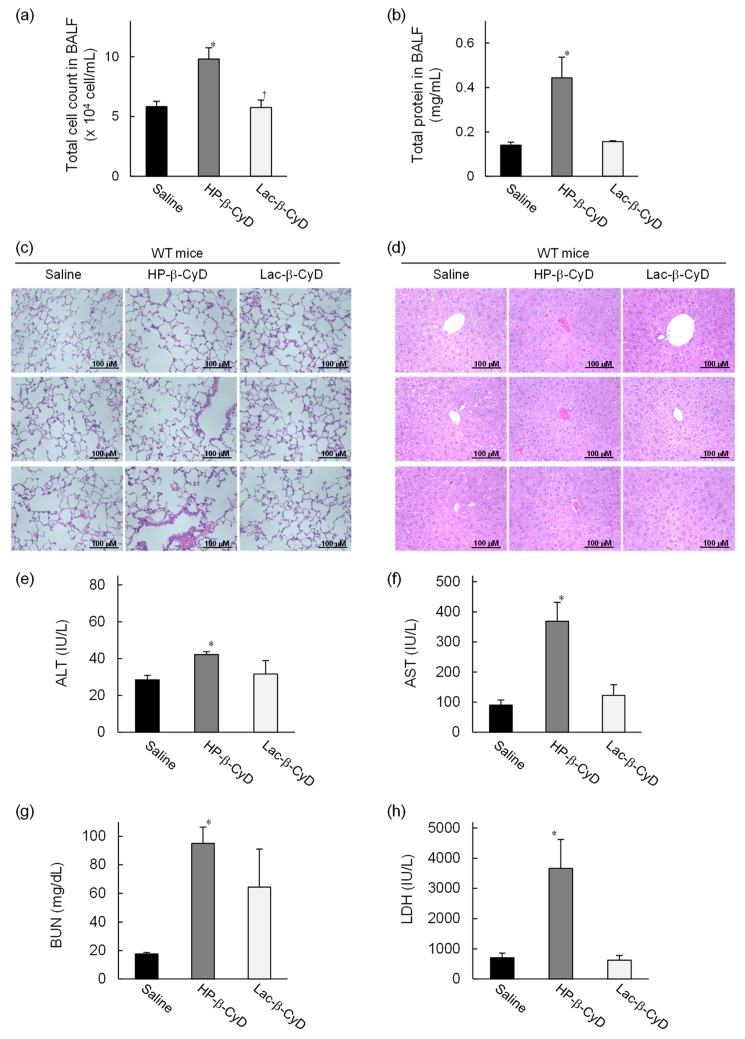
In vivo safety evaluation of Lac-β-CyD after high dose administration to WT mice. Lac-β-CyD (8 mmol/kg, 23,632 mg/kg) or HP-β-CyD (8 mmol/kg, 11,120 mg/kg) was administered to eight-week-old WT mice via a single subcutaneous injection. Blood and organs were collected 8 h after the injection. Lung toxicity was evaluated by (**a**) total cell count in BALF, (**b**) total protein concentration in BALF, and (**c**) histological analysis of the lungs. Liver toxicity was evaluated by (**d**) histological analysis of the liver, and (**e**) AST and (**f**) ALT levels in the plasma. Renal injury and acute toxicity were evaluated by BUN (**g**) and LDH (**h**) levels in plasma, respectively. These figures show the representative images of three different mice (**c**,**d**). Each value represents the mean ± S.E.M. of 4-8 experiments. * *p* < 0.05, compared to control (saline-treated) WT mice.

To compare the side effects of Lac-β-CyD and HP-β-CyD in vivo, their effects to the lungs and livers of WT mice after high dose treatment were evaluated. Lac-β-CyD or HP-β-CyD was administered to eight-week old WT mice at 8 mmol/kg, a dose 11 times higher than the therapeutic dose (Figure 2 and Figure 3). After 8 h, the total cell count and total protein concentration in bronchoalveolar lavage fluid (BALF) were analyzed, histopathological effects on lungs and liver were assessed, and blood levels of AST, ALT, BUN, and LDH were measured (Figure 4). As shown in Figure 4a, treatment with a high dose of HP-β-CyD significantly increased the total cell count in BALF, including inflammatory cells, an indicator of pulmonary toxicity. In contrast, there was no significant difference between the control group and the high dose Lac-β-CyD group. Similarly, the total protein concentration in BALF was significantly increased by a high dose treatment of HP-β-CyD. Meanwhile, the total protein concentration in BALF in the Lac-β-CyD group was comparable to that in the control group.

Histologically it could be seen that treatment with the high dose of HP-β-CyD induced severe hemorrhage, infiltration of inflammatory cells, and a thickened alveolar septum in the lungs of WT mice (Figure 4c). In sharp contrast, the histological sections of the lungs of the control (saline-treated) or high dose Lac-β-CyD-treated mice exhibited a normal morphology.

Although Lac-β-CyD was much less toxic to the lungs than HP-β-CyD, Lac-β-CyD was greatly accumulated in liver tissue after subcutaneous injection (Figure 1). Therefore, to study the effects of a high dose of Lac-β-CyD on the liver, we examined histological changes in liver tissues, and assessed plasma ALT levels. As shown in Figure 4d, significant extensive hepatocellular necrosis and shortages in the glycogen pool were not observed in liver sections after high dose HP-β-CyD and Lac-β-CyD treatments. In addition, treatment with a high dose of HP-β-CyD slightly increased plasma ALT levels, compared to saline (control) or Lac-β-CyD treatment (Figure 4e). Meanwhile, plasma AST levels after treatment with HP-β-CyD drastically increased, compared to saline (control) or Lac-β-CyD treatment, probably due to lung injury (Figure 4f). These data suggest that Lac-β-CyD is far less toxic to the lung than HP-β-CyD.

Parent β-CyD may cause renal toxicity after parenteral administration due to its inherent low solubility. Therefore, parent β-CyD is not suitable for systemic administration. To investigate whether Lac-β-CyD causes renal injury or acute toxicity, we determined the plasma levels of BUN and LDH. As shown in Figure 4g, treatment with a high dose of HP-β-CyD markedly increased BUN levels, compared to saline (control), indicating the induction of renal injury in WT mice, although BUN levels in Lac-β-CyD-treated mice were lower than those in HP-β-CyD-treated mice. In addition, a high dose of HP-β-CyD significantly increased LDH levels compared to saline (control) and that of Lac-β-CyD. Taken together, these results suggest that Lac-β-CyD is likely to have lower toxicity than parent β-CyD and HP-β-CyD, and promising, therefore, for a safer drug candidate for NPC treatment. 

### 3.4. Intracellular Distribution of HF647-Lac-β-CyD in NPC-like Liver Cells

**Figure 5:**
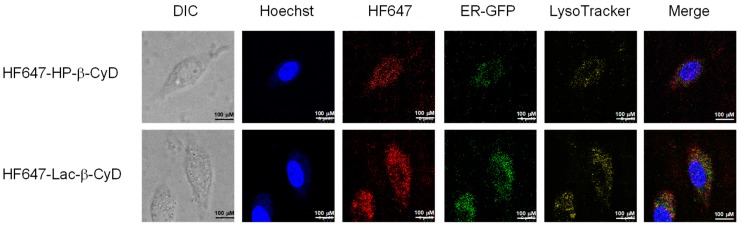
Intracellular distribution of HF647-Lac-β-CyD in NPC-like liver cells. U18666A-treated HepG2 cells were stained by CellLight^®^ ER-GFP, BacMam 2.0 for 16 h at 37 °C. After washing in PBS, the cells were exposed to culture medium containing HF647-Lac-β-CyD (0.1 mM, 24 h) or HF647-HP-β-CyD (0.1 mM, 24 h) with or without AF (1 mg/mL), and were stained using LysoTracker and Hoechst 33342, for endolysosomes and nucleus, respectively. These figures show the representative images of three experiments.

CyDs have a hydrophilic outer surface and a relatively high molecular weight (> ca. 1000 Da), so their cellular uptake is usually very low. Recently, Okada et al. reported that there was some internalization of 1 mM 6-*O*-α-maltosyl-β-CyD into NPC1-deficient CHO cells (0.04 nmol/10^6^ cells; approximately 0.002%) [30]. Previously, we reported that mono-lactose-labeled β-CyD was efficiently internalized into NPC-like liver cells [17]. Therefore, in this study we examined the intracellular distribution of Lac-β-CyD in U18666A-treated HepG2 (ASGPR-positive) cells as a model of ASGPR-expressing NPC-like liver cells. Here, we used U18666A, an inhibitor of intracellular cholesterol trafficking, to induce the NPC phenotype [31]. The intracellular uptake of HF647-Lac-β-CyD into NPC-like liver cells was higher than that of HF647-HP-β-CyD (Figure 5). 

In NPC cells, there is a defect in cholesterol trafficking from the endolysosomes to the endoplasmic reticulum (ER), resulting in cholesterol accumulation in the endolysosomes. Therefore, the subcellular localization of Lac-β-CyD is another crucial factor for determining its therapeutic efficacy in NPC disease. To investigate the subcellular localization of HF647-Lac-β-CyD in the NPC-like liver cells, the endolysosomes and ER were stained with specific indicators, LysoTracker^®^ and ER-GFP, respectively, and the resulting fluorescence was observed by confocal laser scanning microscopy. Notably, most of the intracellular HF647-Lac-β-CyD was colocalized with endolysosomes (Figure 5; shown in yellow). Furthermore, some colocalization of HF647-Lac-β-CyD with both endolysosomes and ER was observed, suggesting that Lac-β-CyD may be distributed in ER-endolysosome membrane contact sites (Figure 5). Collectively, the data indicate that Lac-β-CyD enters the NPC-like liver cells via ASGPR-medicated endocytosis and may be distributed to the endolysosomes and ER. 

### 3.5. Free Cholesterol Lowering Effect of Lac-β-CyD in Endolysosomes

**Figure 6:**
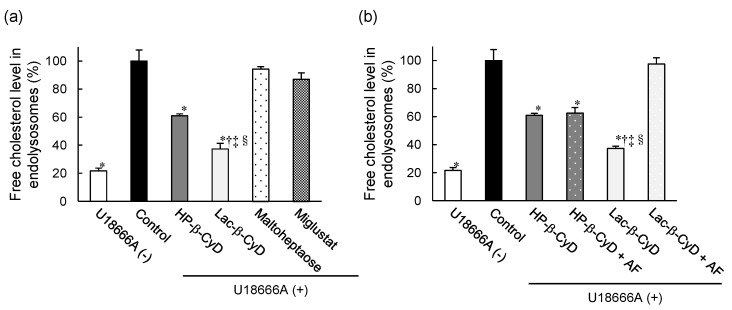
Free cholesterol-lowering effect of Lac-β-CyD in NPC-like liver cells. (**a**) U18666A-treated HepG2 cells were incubated with culture medium containing Lac-β-CyD, HP-β-CyD, miglustat, or maltoheptaose (1 mM, 24 h) at 37 °C, and fixed in 4% PFA. Fluorescence of cells stained with Filipin III (cholesterol) and LysoTracker^®^ Green DND-26 (endolysosomes). Quantification of Filipin III fluorescence intensity in endolysosomes using ImageJ software. Each value represents the mean ± S.E.M. of 3 experiments. * *p* < 0.05, compared to control; † *p* < 0.05, compared to HP-β-CyD; ‡ *p* < 0.05, compared to maltoheptaose; § *p* < 0.05, compared to miglustat. (**b**) Effect of AF on the Lac-β-CyD free cholesterol lowering effect in endolysosomes of U18666A-treated HepG2 cells. Cells were exposed to culture medium containing Lac-β-CyD or HP-β-CyD (1 mM) ± AF (1.0 mg/mL) for 24 h at 37 °C. Fluorescence of cells stained with Filipin III (cholesterol) and LysoTracker^®^ Green DND-26 (endolysosomes). Quantification of Filipin III fluorescence intensity in endolysosomes using ImageJ software. Each value represents the mean ± S.E.M. of 3 experiments. * *p* < 0.05, compared to control; † *p* < 0.05, compared to HP-β-CyD; ‡ *p* < 0.05, compared to HP-β-CyD + AF; § *p* < 0.05, compared to Lac-β-CyD + AF.

We examined the effect of Lac-β-CyD on free cholesterol accumulation in endolysosomes of NPC model cells by Filipin III staining, a fluorescent cholesterol binding molecule (Supplementary Figure S1, Figure 6). As shown in Supplementary Figure S1a, control NPC-like liver cells treated with U18666A clearly exhibited higher Filipin III fluorescence signals than normal untreated cells. In addition, the Filipin III fluorescence signals were co-localized with LysoTracker^®^ fluorescence signals, suggesting that free cholesterol accumulates in endolysosomes of control NPC-like liver cells. Figure 6a shows the quantitative intensity data from Filipin III fluorescence images in Figure 6a. Treatment with 1 mM Lac-β-CyD or HP-β-CyD for 24 h drastically reduced the fluorescent intensity of Filipin III in NPC-like liver cells, compared to that of the control cells (Supplementary Figure S1a, Figure 6a). Notably, the free cholesterol lowering effect of Lac-β-CyD was significantly stronger than that of HP-β-CyD after 24 h (Figure 6a). To further characterize the cholesterol lowering effects of Lac-β-CyD and HP-β-CyD, we treated NPC-like liver cells with maltoheptaose, which has a linear chain structure of seven glucose moieties. Interestingly, treatment with 1 mM maltoheptaose did not change the fluorescence intensities of Filipin III after Lac-β-CyD or HP-β-CyD treatment, compared to that of control. In addition, treatment with 1 mM miglustat, which is a commercial drug for the treatment of NPC patients, also did not lower the fluorescence signals of Filipin III under these experimental conditions (Figure 6a). This may be due to the different mechanisms between miglustat, a glycosylceramide synthase inhibitor, and Lac-β-CyD for NPC treatment.

Additionally, to investigate the involvement of ASGPR in the cholesterol-lowering effect of Lac-β-CyD, we examined Filipin III fluorescence after Lac-β-CyD or HP-β-CyD treatment in the presence or absence of AF (Supplementary Figure S1b, Figure 6b). The cholesterol lowering effect of Lac-β-CyD was drastically impaired by co-treatment with AF, indicating that ASGPR-mediated endocytosis of Lac-β-CyD is important to lower free cholesterol levels in endolysosomes of the cells. On the other hand, the intensity of Filipin III fluorescence after treatment with HP-β-CyD did not change following the co-treatment of HP-β-CyD with AF. Based on these results, we postulate that Lac-β-CyD decreases the accumulation of free cholesterol in endolysosomes of NPC-like liver cells via ASGPR-mediated endocytosis. 

### 3.6. In Vitro Safety Evaluation of Lac-*β*-CyD

**Figure 7:**
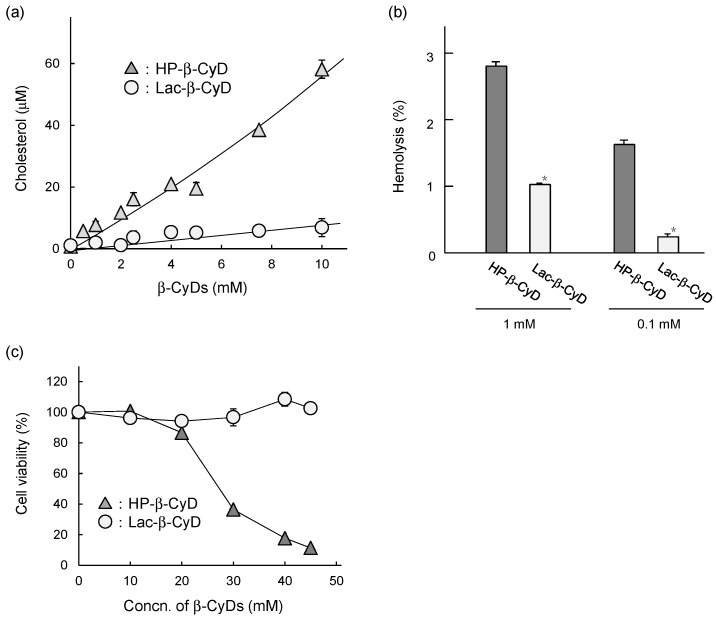
In vitro safety evaluation of Lac-β-CyD. (**a**) Phase solubility diagram of cholesterol with Lac-β-CyD and HP-β-CyD in water at room temperature. (**b**) Hemolytic activity of Lac-β-CyD and HP-β-CyD (at 0.1 and 1.0 mM) in rabbit red blood cells. (**c**) Cytotoxicity of Lac-β-CyD and HP-β-CyD in U18666A-treated HepG2 cells. Cell viability was determined by WST-8 assay. Each value represents the mean ± S.E.M. of 4 experiments. * *p* < 0.05, compared to HP-β-CyD.

It has been reported that the cytotoxic and hemolytic activities of HP-β-CyD are correlated with its cholesterol-solubilizing capacities. To examine the interaction of Lac-β-CyD with cholesterol, we performed a phase solubility study. Figure 7a shows the phase solubility diagrams for cholesterol with Lac-β-CyD or HP-β-CyD in water at 25 °C. The solubility of cholesterol increased almost linearly as a function of the Lac-β-CyD concentration, suggesting the formation of a soluble complex of cholesterol with Lac-β-CyD at a 1:1 molar ratio. Meanwhile, the solubility of cholesterol increased with an upward deviation from linearity as the concentration of HP-β-CyD increased, suggesting the formation of higher-order complexes, at a molar ratio of 1:1 and 1:2 (cholesterol:HP-β-CyD). These results suggest that although both compounds can solubilize cholesterol, HP-β-CyD has a stronger interaction with cholesterol than Lac-β-CyD. We previously reported that HP-β-CyD induces hemolysis and cytotoxicity at high concentrations via the extraction of cholesterol from the plasma membranes of cells [12,32]. As toxic hemolysis has a major impact on patient safety, we studied the hemolytic activity of Lac-β-CyD on rabbit red blood cells (Figure 7b). Treatment with 0.1 mM or 1 mM Lac-β-CyD resulted in significantly lower hemolytic activity than that with HP-β-CyD at the same concentrations. Finally, we examined the cytotoxicity of Lac-β-CyD in NPC-like liver cells (Figure 7c). Treatment with HP-β-CyD resulted in a concentration-dependent decrease in cell viability, whereas treatment with Lac-β-CyD, up to 45 mM, had no effect on cell viability. These results suggest that Lac-β-CyD has lower cytotoxicity than HP-β-CyD due to its weaker interaction with cholesterol. 

## 4. Discussion

In this study, the therapeutic effects of Lac-β-CyD on hepatomegaly in NPC model mice, and its safety in WT mice, were evaluated. Our in vivo fluorescence imaging analysis revealed that the accumulation of HF647-Lac-β-CyD in liver tissue after subcutaneous administration was significantly higher than that of HF647-HP-β-CyD (Figure 1a). In addition, the accumulation of HF647-Lac-β-CyD was inhibited in the presence of AF, which is a competitive inhibitor of ASGPR, suggesting that Lac-β-CyD might accumulate in liver tissue through its interaction with ASGPR.

HF647-Lac-β-CyD also accumulated in the kidney. In general, water-soluble molecules, with a molecular weight <45,000 Da which are not bound to plasma protein are freely filtered by the glomerulus, resulting in renal excretion. Previously, Frijlink et al. reported that HP-β-CyD accumulates mainly in the kidney after subcutaneous injection, because of rapid renal clearance [32]. Therefore, accumulation of Lac-β-CyD in the kidney may also be due to renal clearance. Histological analysis and determination of BUN levels in *Npc1*^−/−^ mice and WT mice after subcutaneous administration of Lac-β-CyD (Figure 2a, Figure 3a and Figure 4g) indicated that Lac-β-CyD did not cause any significant toxicological effects on the kidney. Furthermore, Lac-β-CyD had a weak interaction with cholesterol (Figure 7a) and low cytotoxicity in NPC-like liver cells (Figure 7c), suggesting that Lac-β-CyD is a safer drug candidate for NPC treatment. Unfortunately, no studies have been conducted on the integrity and functional status of the regional lymph nodes following the subcutaneous injection of Lac-β-CyD, nor on Kupffer cell and splenic macrophage function. Further elaborate studies on these biological functions are necessary to expand the application of Lac-β-CyD to clinical stage.

The *Npc1*^−/−^ mice exhibit various phenotypes, such as vacuolization of several tissues and elevation of ALT as hepatic injury markers. In our study, a number of vacuoles were observed in histological sections of the liver tissue of *Npc1*^−/−^ mice, probably due to free cholesterol accumulation in hepatocytes (Figure 2 and Figure 3). Lac-β-CyD lowered free cholesterol accumulation in the hepatocytes (Figure 2d and Figure 3d), resulting in a decreased number of vacuoles in the liver tissue (Figure 2a and Figure 3a) and an improvement in the hepatic injury markers of ALT (Figure 2b,c and Figure 3b,c). In addition, we demonstrated that Lac-β-CyD accumulated in the liver after subcutaneous administration (Figure 1). Furthermore, our in vitro cellular uptake study suggests that Lac-β-CyD can enter the hepatocytes through its interaction with ASGPR, specifically expressed on hepatocytes (Figure 5). We postulate that Lac-β-CyD reduces vacuolization in the liver tissue of *Npc1*^−/−^ mice due to its cholesterol lowering effect on hepatocytes after uptake into the cells. Meanwhile, these results may correlate with toxicity reported in Figure 7. It can be thought that 8 mmol/kg of HP-β-CyD is more toxic than Lac-β-CyD but maybe HP-β-CyD is more efficient to trap the membrane cholesterol. 

This hepatocyte-selective cellular uptake may enable a reduction in the dose of Lac-β-CyD required for effective treatment of hepatomegaly in NPC. We demonstrated that Lac-β-CyD enters hepatocytes by clathrin-mediated endocytosis via ASGPR, and is promptly internalized into endolysosomes in NPC-like liver cells, in contrast to HP-β-CyD (Figure 5). This suggests that a lower dose of Lac-β-CyD than that of HP-β-CyD may be possible for the treatment of hepatomegaly in NPC. However, the mechanism underlying the free cholesterol lowering effect of Lac-β-CyD is still unclear. In general, ligands internalized via ASGPR or LDL receptor-mediated endocytosis are dissociated when protons flow into the endosomes at a pH range of 6.0 to 6.5. The dissociated receptors are recycled and the ligands reach the endolysosomes [33,34]. Furthermore, it has been reported that 6-*O*-α-maltosyl-β-CyD is slightly internalized into endolysosomes in NPC model cells, and is released to the extracellular fluid as a complex of 6-*O*-α-maltosyl-β-CyD and free cholesterol [30]. Therefore, it may be possible that after internalization of Lac-β-CyD into hepatocytes by ASGPR-mediated endocytosis, Lac-β-CyD may dissociate from ASGPR and solubilize cholesterol in endolysosomes through complex formation, then finally exit from endolysosomes as a complex with cholesterol. Notably, we found that Lac-β-CyD was partially co-localized with both endolysosomes and the endoplasmic reticulum (ER) (Figure 5). Recently, Kant et al. demonstrated that cholesterol transfer from endosomes to the ER and other organelles is predominantly mediated by lipid-binding proteins that localize at membrane contact sites [35]. In addition, it is reported that free cholesterol in endolysosomes is transferred to the ER via membrane contact sites and is esterified by acyl-CoA cholesterol acyltransferase (ACAT) [36,37]. However, in NPC bearing a spontaneous mutation of the *Npc1* or *Npc2* gene, there is a defect in cholesterol trafficking from the endolysosomes to the ER. Therefore, Lac-β-CyD may improve cholesterol transfer from the endolysosomes to the ER via membrane contact sites. Further elaborate studies are necessary to elucidate not only the role of Lac-β-CyD in cholesterol transfer in cells but also the ability of the Lac-β-CyD to trap the cellular cholesterol located to the membrane.

## 5. Conclusions

**Figure 8:**
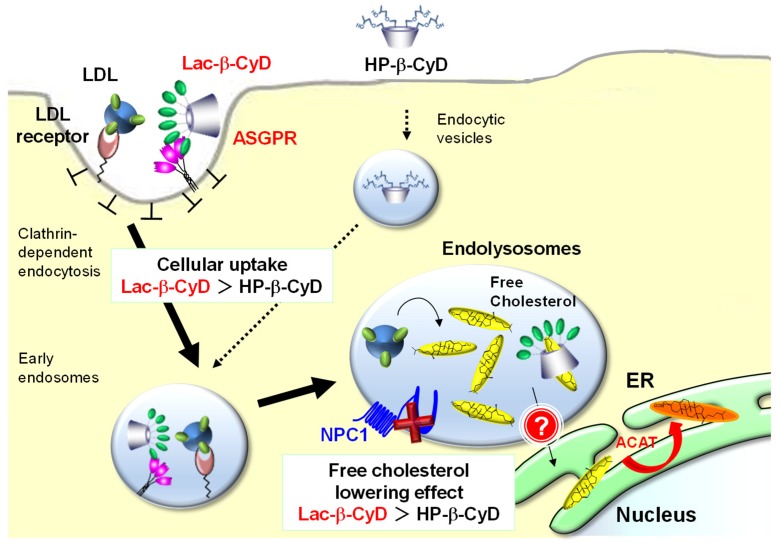
Proposed mechanism of treatment efficacy of Lac-β-CyD for hepatomegaly in NPC.

In the present study, we demonstrated that Lac-β-CyD mainly accumulates in the liver via ASGPR-mediated endocytosis in NPC model mice after subcutaneous administration. In addition, Lac-β-CyD significantly lowered free cholesterol levels in endolysosomes of hepatocytes, and improved hepatomegaly in NPC model mice. Notably, a high dose of Lac-β-CyD did not show the side effects seen after HP-β-CyD administration. Our findings suggest that Lac-β-CyD may have potential as a treatment for hepatomegaly in NPC (Figure 8).

## Supplementary Materials

The following are available online at https://www.mdpi.com/2079-4991/9/5/802/s1, Figure S1: Free cholesterol-lowering effect of Lac-β-CyD in NPC-like liver cells.
