# Supplementary material for: In vivo Efficacy and Safety Evaluation of Lactosyl-β-cyclodextrin as a Therapeutic Agent for Hepatomegaly in Niemann-Pick Type C Disease
Source: Nanomaterials (Basel). 2019 May 25;9(5):802. doi: 10.3390/nano9050802 (PMC6566927; doi:10.3390/nano9050802)
Supplement: Supplementary file 1 [file nanomaterials-09-00802-s001.pdf]

Supplementary material

In vivo Efficacy and Safety Evaluation of Lactosyl-β-cyclodextrin as a Therapeutic Agent for Hepatomegaly in Niemann-Pick Type C Disease

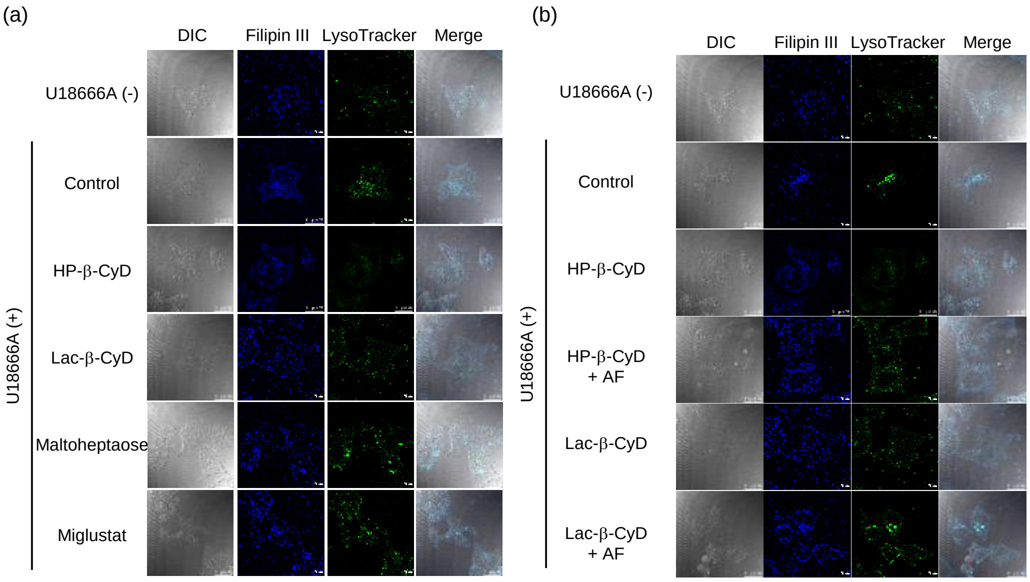

Figure S1. Free cholesterol-lowering effect of Lac-β-CyD in NPC-like liver cells. U18666A-treated HepG2 cells were incubated with culture medium containing Lac-β-CyD, HP-β-CyD, miglustat, or maltoheptaose (1 mM, 24 h) at 37°C, and fixed in 4% PFA. (a) Fluorescence of cells stained with Filipin III (cholesterol) and LysoTracker® Green DND-26 (endolysosomes). Effect of AF on the Lac-β-CyD free cholesterol lowering effect in endolysosomes of U18666A-treated HepG2 cells. Cells were exposed to culture medium containing Lac-β-CyD or HP-β-CyD (1 mM) ± AF (1.0 mg/mL) for 24 h at 37 °C. (b) Fluorescence of cells stained with Filipin III (cholesterol) and LysoTracker® Green DND-26 (endolysosomes).
